# The Value of Family History in Diagnosing Primary Immunodeficiency Disorders

**DOI:** 10.1155/2014/516256

**Published:** 2014-08-05

**Authors:** Mohamed A. Hendaus, Ahmad Alhammadi, Mehdi M. Adeli, Fawzia Al-Yafei

**Affiliations:** ^1^Department of Pediatrics, Section of General Pediatrics, Hamad Medical Corporation, Doha, Qatar; ^2^Weill Cornell Medical College, Doha, Qatar; ^3^Department of Pediatrics, Allergy and Immunology Section, Hamad Medical Corporation, Doha, Qatar; ^4^Department of Pediatrics, Endocrinology Section, Hamad Medical Corporation, Doha, Qatar

## Abstract

Eliciting proper family medical history is critical in decreasing morbidity and mortality in patients with primary immunodeficiency disorders (PIDs). Communities with a common practice of consanguinity have a high rate of PIDs. We are presenting 2 cases where digging deeply into the family medical history resulted in the diagnosis of Omenn syndrome, a possibly fatal entity if not managed in a reasonable period.

## 1. Introduction

Primary immunodeficiency disorders (PIDs) usually refer to various genetic disorders that disturb fundamental parts of the immune system resulting in flaws in function, differentiation, or both of these parts [1]. Early diagnosis of PID is crucial because often lifesaving interventions can be provided to decrease the rate of morbidity and mortality [2]. A major proportion of affected infants and children are offspring of consanguineous marriage; in addition, family medical history is very common in PID and it is an essential component in the diagnosis [3].

We are presenting two cases that show how crucial eliciting proper family medical history is in saving lives of infants and children with PID.

## 2. Case #1

A 3-month-old Pakistani female presented with generalized rash and failure to gain appropriate weight. The infant was born full term via normal spontaneous vaginal delivery. The initial family history was unremarkable except that parents are first degree cousins. The patient was admitted for failure to thrive workup. On examination, the vitals were appropriate for age. Anthropometric measurements were normal for age except the weight was below the 3rd percentile. The patient looked nontoxic, but thin. There was a generalized maculopapular rash with two bullae on the gluteal area. The rest of the examination was unremarkable. The rash disappeared on the second day of admission. Laboratory investigations showed hemoglobin of 6.6 g/dL, positive direct Coombs test, and positive occult blood.

The differential diagnosis was cow milk allergy, autoimmune hemolytic anemia, and sepsis.

We decided to transfuse the patient with packed red blood cells (PRBCs) due to severe anemia and premedicated the patient with diphenhydramine. Severe diffuse erythema and irritability developed and we had to discontinue the transfusion process. We reassessed the family medical history and the father mentioned that two older siblings passed away while receiving PRBC for the same condition. The new family medical history prompted us to broaden our laboratory investigation.

Her lab results showed white blood count (WBC) of 5400/uL, decreased absolute lymphocyte count (ALC) 2300/uL, increased immunoglobulin E (IgE) 213 Ku/L, IgA (98 mg/dL), and IgM (275 mg/dL). The lymphocyte subpopulation showed CD4 lymphopenia (212 cells/uL) and significant reduction in B cells (193 cells/uL) with normal NK cells count (1211 cells/uL); the majority of T cells receptors showed abnormal gamma/delta receptors, which can be seen in Omenn syndrome and hypomorphic RAGI/II mutation. In addition, the T cell function assay was abnormally low (below 15% of control). All of this confirmed the diagnosis of SCID variant.

## 3. Case #2

A 3-month-old Qatari female was admitted due to generalized rash, lymphadenopathy, and hepatosplenomegaly diagnosed by her pediatrician. The infant was born full term via normal spontaneous vaginal delivery. The initial family history was unremarkable except that parents are first degree cousins. On examination, the vitals were appropriate for age as well as the anthropometric measurements. There was a generalized maculopapular rash. In addition there were bilateral, nontender, nonerythematous axillary lymph nodes with diameter of 0.5 cm. The liver and spleen were palpable 3 cm below the costal margin. The rest of the examination was unremarkable. On further family medical history reassessment, it showed that both parents are carriers of Omenn syndrome and two siblings are affected by the disease.

Laboratory investigation showed that our patient had WBC of 9500/uL, lymphopenia (ALC: 600 cells/uL) with low T cells (7 cells/uL), very low both CD4 (4 cells/uL) and CD8 (2 cells/uL), absent B cells (0.00 cells/uL), and normal NK cells count (133 cells/uL) in the lymphocyte subpopulation; T cell function test showed no response to mitogens. In addition, the immunoglobulins levels showed low IgA (<5 mg/dL) and IgM (<4 mg/dL) with high IgE for age (2 Ku/L); all this confirmed the diagnosis of SCID variant.

Both cases were sent for bone marrow transplant (BMT) abroad due to the unavailability of those services in our institution.

## 4. Discussion

PIDs are acquired by different modes of inheritance [4]. Communities with a common practice of consanguinity, such as Iran, Saudi Arabia, Turkey, Morocco, Egypt, Kuwait, and Oman, have a high rate of PIDs [3]. In addition, autosomal recessive inheritance is not the only culprit in consanguineous marriage, but multifactorial factors play a role as well [5]. In infants and children with PID, a family history is positive in roughly 66% of all patients, with the highest rate in immune dysregulation (up to 100%) and phagocyte defects (92%) [3].

Family history is one of the components of the ten warning signs of PIDs as reported by Jeffrey Modell Foundation (JMF) [6]. Arkwright and Gennery studied the validity of these warning signs and found that family history is a strong identifier in PID [7]. Eliciting proper family history of PIDs is known to be crucial in avoiding delay in diagnosis and hence morbidity and mortality [8].

In a large study conducted by Rezaei et al., it was found that 65.6% of PID patients were the results of consanguineous marriages. Consanguinity was found to be 75.8% in combined immunodeficiency, 77.8% in cellular immunodeficiency, 72.5% in defects of phagocytic function, 58.6% in other immunodeficiencies, 54.1% in predominantly antibody deficiency, and 50% in complement deficiency [9].

Omenn syndrome is an autosomal recessive disorder that presents with lymphocytic infiltration of the gut, liver, spleen, and skin that results in skin changes similar to graft-versus-host disease; in addition, affected children present with diarrhea and failure to thrive [10].

The majority of mutations in Omenn syndrome are missense mutations in recombinase activating genes RAG1 and RAG2. Those mutations are usually detected on chromosome 11 [11, 12].

Children with Omenn syndrome usually present with hypogammaglobulinemia and elevated serum immunoglobulin E levels [10]. Moreover, affected children usually have absent B cells with present natural killers (NK) cells. The T lymphocytes count might be normal to high with variable distribution ratio of CD4+ : CD8+ subsets [13].

All children with Omenn syndrome have a high mortality rate in the first six months of age unless allogeneic haematopoietic stem cell transplantation is offered [10].

Our patients' parents perceived consanguinity and family medical history as unremarkable. Parent's perception might be different from the physician's. Moreover, people perceive family medical history differently and hence might be misleading. Hunt et al. stated that some individuals required a large number of affected relatives to perceive that they have a substantial family medical history [14].

Since family medical history could be lifesaving, some authors advocate for going one step further and interview additional family members, review death certificates, autopsy reports, and even review family members' medical reports [15].

Primary care physicians (PCPs) do not always elicit good family medical history and it is usually attributed to lack of time [16]. In addition, PCPs allot less than the suggested time to elicit family history information [15]. Other barriers, as perceived by PCPs, are lack of skills to take appropriate family medical history and counseling [17, 18] and lack of knowledge in the genetic field to merge up-to-date medical information in their practice [19]. Our team members reconsidered the initial elicited family medical history per patients' clinical course. More detailed history and definite determination by physicians were indispensable in the diagnoses of our patients.

## 5. Conclusion

Eliciting proper family medical history might be crucial in decreasing morbidity and mortality in infants and children. It is very important to educate communities that consanguineous marriage might be detrimental for the health of new generation. In addition, premarital counseling as well as newborn screening is recommended.
